# Effects of Cavity Structure on Tuning Properties of Polymer Lasers in a Liquid Environment

**DOI:** 10.3390/polym11020329

**Published:** 2019-02-14

**Authors:** Fengzhao Cao, Shuai Zhang, Junhua Tong, Chao Chen, Lianze Niu, Tianrui Zhai, Xinping Zhang

**Affiliations:** Institute of Information Photonics Technology and College of Applied Sciences, Beijing University of Technology, Beijing 100124, China; wincfz@163.com (F.C.); 13844225221@163.com (S.Z.); jhtong@emails.bjut.edu.cn (J.T.); s201706083@emails.bjut.edu.cn (C.C.); niulianze@126.com (L.N.); zhangxinping@bjut.edu.cn (X.Z.)

**Keywords:** tunability, polymer lasers, distributed feedback, cavity structure

## Abstract

The effect of cavity structures on the tuning properties of polymer lasers was investigated in two common distributed-feedback cavities. The configurations of the two cavities are substrate/grating/active waveguide and substrate/active waveguide/grating, respectively. The polymer lasers were operated in the liquid environment, and the laser wavelength was tuned dynamically by changing the refractive index of the liquid. Polymer lasers based on the substrate/grating/active waveguide structure showed a higher tunability than those based on the substrate/active waveguide/grating structure due to a larger electric field distribution of the laser mode in the liquid environment. It is expected that these results will be useful in the development of tunable laser sources.

## 1. Introduction

The polymer material is an ideal laser material due to the advantages of broad photoluminescence (PL) spectra in the visible region and good film-forming property [1,2]. Distributed feedback (DFB) lasers based on polymer materials have attracted wide attention because their wavelength tunability offers greater possibilities for the lasers’ practical applications. DFB polymer lasers have obvious advantages, including (1) the fact that various kinds of fabrication techniques can be used to fabricate DFB polymer lasers such as nanoimprint [3,4,5], direct writing [6,7], electron beam etching [8], reaction ion beam etching [9], and interference etching [10]. (2) DFB polymer lasers can be fabricated on the flexible substrates [11] and the fiber facets [12]. (3) The threshold of DFB polymer lasers is relatively lower than the other configurations [13,14]. Moreover, the possibility of using laser diodes or light-emitting diodes as optical pumps has been proposed [15,16,17], which is useful for practical applications. 

In recent years, the tunability of DFB polymer lasers had been demonstrated by many approaches. These studies focused primarily on achieving a wavelength tunability by varying the period or the refractive index, such as wedge-shaped layers [18,19], chirped or segmented cavities [11,20,21], electric directed reconfiguration [22], compound cavity [23,24], and so on. But most of the methods only varied statically the laser wavelength. In other words, the laser wavelength is unchanged if the excited area of the polymer laser is fixed. However, some research groups have started to investigate the dynamic tunability of the emission of polymer lasers. By employing the flexible materials, the laser wavelength can be tuned dynamically by bending or stretching the laser devices [6,25,26]. Moreover, the wavelength of DFB polymer lasers is sensitive to environmental parameters such as temperature, pressure, and refractive index. So, the wavelength of polymer lasers can also be tuned dynamically by changing the environmental parameters. Thermally-tunable liquid crystals have been used to change the ambient refractive index of the polymer laser [27]. 

**Figure 1:**
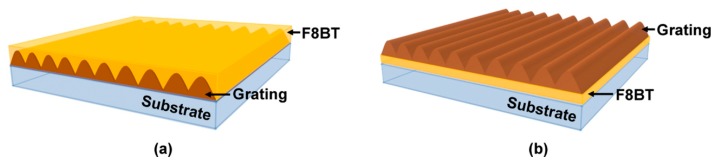
Schematic diagram of distributed feedback (DFB) polymer lasers based on (**a**) substrate/grating/active waveguide (SGA) structure and (**b**) substrate/active waveguide/grating (SAG) structure.

This paper explored the tunability of the DFB polymer lasers by changing the ambient refractive index. Based on two common DFB cavities, the influence of cavity structures on the tunability of polymer lasers was investigated systematically. One configuration was a substrate/grating/active waveguide (SGA) structure, and the other was a substrate/active waveguide/grating (SAG) structure, as shown in Figure 1. In the experiment, the laser device was sealed in a chamber and filled with a circulating liquid. The laser wavelength can be tuned by changing the refractive index of the liquid. Polymer lasers based on the SGA structure showed a higher tunability than those based on the SAG structure. It can be attributed to a large electric field distribution of the laser mode in the liquid environment for the SGA structure. 

## 2. Fabrication of the SGA and SAG Cavities

Figure 1 showed the schematic diagram of the DFB polymer lasers based on the SGA and SAG structure. In the experiment, the two-beam interference method was used to fabricate the grating structure. For the SGA structure, the photoresist was spin coated onto a glass substrate at 2000 rpm, forming a 110 nm film. Then the film was heated on a hot plate at 110 °C for 1 minute. A diode-pumped solid-state laser (FLARE NX, Coherent, Santa Clara, CA, USA) was used to fabricate the grating structure, which had a wavelength of 343 nm, a pulse width of 1 ns, and a repetition rate of 500 Hz. After exposure to a two-beam interference pattern, the sample was developed for 5 seconds and stopped with deionized water. After heating on a hot plate for 1 minute to remove the redundant solvent, the gain material poly[(9,9-dioctylfluorenyl-2,7-diyl)-alt-*co*-(1,4-benzo-{2,1′,3}-thiadiazole)] (F8BT, American Dye Source, Monteral, QC, Canada) was spin-coated on the grating. The concentration of F8BT solution in xylene was 23 mg/ml. The spin speed was 1500 rpm, and the film thickness was 150 nm. For the SAG structure, the process parameters were identical to the SGA structure, and only the sequence was changed. The gain material was first spin-coated on the glass substrate. The sample was heated on a hot plate for 1 minute to remove the solvent. Then the photoresist was spin-coated on the gain materials. Finally, a grating was fabricated on the gain materials, forming a SAG structure. The period of the grating was 335 nm. The depth of the grating was about 110 nm.

**Figure 2:**
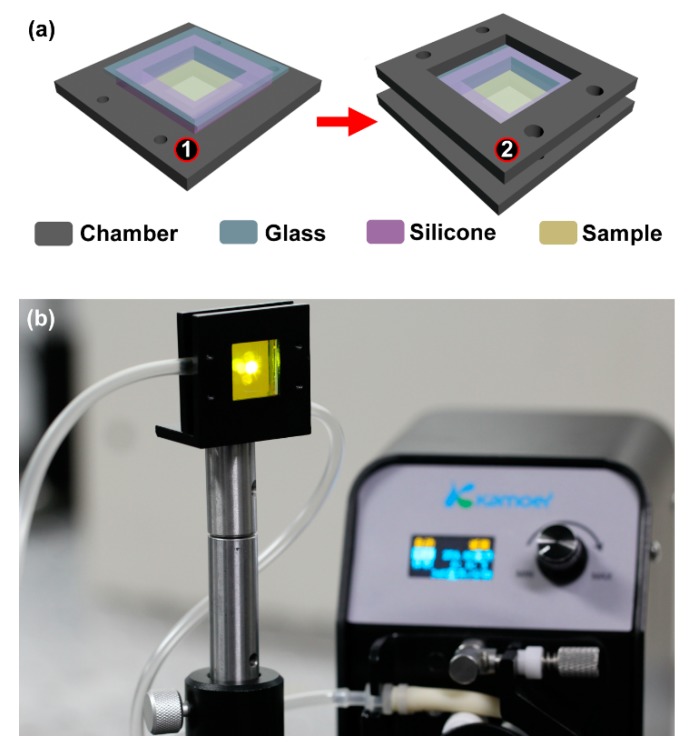
(**a**) Schematic of the sealed sample chamber. The gray, blue, pink, and yellow rectangles represent the sample chamber, glass coating, silicone, and the laser sample, respectively. (**b**) Photograph of the measurement system.

Figure 2a presented a schematic diagram of the sealed sample chamber. The sealed sample chamber was designed to fill the liquid and place the sample. The sample chamber consisted of four parts including the two frames (① and ② in Figure 2a), a silicone and a glass coating. Frame ① and frame ② had dimensions of 4.2 cm × 4.2 cm and a thickness of 3 mm. There was a square opening at the center of frame ① with a size of 2.4 cm^2^ and a depth of 1.5 mm. The center of the square opening was hollowed out with a size of 1 cm^2^. The DFB laser was located at the center of the sample chamber. The substrate of the DFB laser was 1.5 cm^2^. The spot of the pump beam was in the center of the sample chamber, as shown in Figure 2b. The chamber was sealed with silicone strips around the DFB polymer laser, and the thickness of the silicone strips was 4 mm. Then, the glass was covered on the silicone strips. Finally, frame ② was assembled with the prepared frame ① to form a sealed sample chamber. The center of frame ② was hollowed out completely, and the area of the hollowed out section was 2.4 cm^2^. The surface of the DFB polymer laser faced the inside of the chamber to contact with the liquid directly. 

The sealed sample chamber was provided with an inlet and an outlet, and the liquid can be circulated by a peristaltic pump. In this work, the liquid was changed to achieve different refractive indices, including deionized water and sucrose solutions of different concentrations (33, 50, and 60%). The wavelength of the DFB polymer laser was tuned dynamically.

The measurement system based on the sealed assembled sample chamber was shown in Figure 2b. The two syringe needles were inserted into the silicone layer on both sides of the sample chamber to form an inlet and an outlet. The channels connected in turn to the tested liquid, the sample chamber, and the peristaltic pump. The liquid with different refractive indices can be circulated by employing the peristaltic pump. 

## 3. Spectra Characterization of the SGA and SAG Cavities

**Figure 3:**
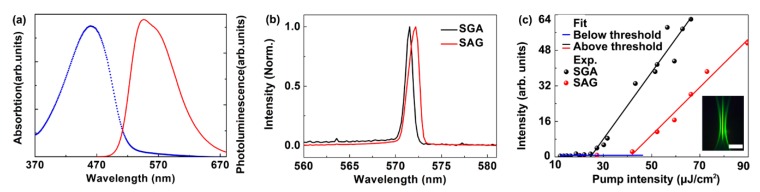
(**a**) The absorption (blue curve) and the photoluminescence (PL) spectra of F8BT (red curve). (**b**) Measured spectra of the lasing emission of the polymer laser based on SGA cavity (black curve) and the SAG cavity (red curve). (**c**) The output intensity as a function of the pump intensity of the distributed feedback (DFB) polymer laser based on SGA cavity (black curve) and the SAG cavity (red curve). The inset demonstrates a typical laser spot when the pump intensity is about 50 µJ/cm^2^. The scale bar is 1 cm.

Figure 3a showed the spectroscopic properties of F8BT. The blue curve in Figure 3a showed that the absorption of F8BT was centered at about 470 nm. The PL spectra were centered at 560 nm as denoted by the red curve. The laser was excited by a frequency-doubled Ti: Sapphire laser operating at 1 kHz, with a wavelength of 400 nm, pulse duration 200 fs and pulse energy up to 1 mJ. The pump power was controlled by a neutral density filter. The pump spot area was approximately 3 mm^2^, which was adjusted by a 15 cm focal-length lens. The pump beam illuminated on the sample at an angle of 20° and the emission laser was collected by a spectrometer (Maya 2000 Pro, Ocean Optics, FL, USA). Figure 3b showed the spectra of the lasing emission of the polymer laser based on SGA cavity (black curve) and the SAG cavity (red curve). The laser wavelength of the SGA cavity and the SAG cavity was located at 571.5 nm and 572.2 nm, respectively. Figure 3c showed the output intensity as a function of the pump intensities. It can be seen that the threshold of the SGA cavity and the SAG cavity is 24.7 µJ/cm^2^ and 43.0 µJ/cm^2^, respectively. The inset in Figure 3c showed the photograph of the transverse mode of the laser spot when the pump intensity exceeded the threshold (~50.0 µJ/cm^2^). The laser spot hit on a white paper. The distance between the laser device and the white paper was 100 mm. The size of the laser spot was about 1 mm × 35 mm. The profile of the laser beam had a symmetric shape and a bright yellow-green laser spot was observed at the center. The shape of the laser pattern was defined by the Bragg diffraction of the cavity.

Generally, there are two classes of feedback mechanisms for DFB lasers, including gain-coupled and refractive index modulation-coupled DFB regimes [28,29]. For the gain-coupled DFB regime, the laser emission is governed by the formula 2n_eff_Λ = mλ, where n_eff_ is the effective refractive index of the DFB mode, Λ is the grating period, m is the diffraction order, and λ is the emission wavelength. In our experiment, the DFB laser was a vertical surface-emitting device. That is to say, m equals 2. For a fixed grating period, the lasing wavelength is influenced by the ambient refractive index, i.e., *λ* = n_eff_Λ.

**Figure 4:**
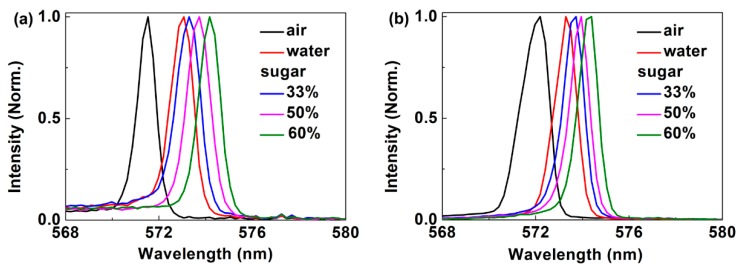
Measured spectra of the DFB polymer laser with different ambient refractive indices. (**a**) The SGA cavity. (**b**) The SAG cavity.

Figure 4 illustrated the tunability of the DFB polymer lasers based on the SGA and SAG cavities. As shown in Figure 4a, the emission wavelength of the polymer laser based on the SGA cavity was tuned with changing the ambient materials from air to different liquids. When the ambient refractive index changed from 1.0 (air) to 1.33 (water), the emission wavelength shifted from 571.5 nm to 573.1 nm. The sucrose solutions with different concentrations were used by dissolving the sugar in the deionized water. The emission wavelength can be tuned by using sucrose solutions with different concentrations, as shown in Figure 4a. The laser wavelength was tuned from 573.3 nm to 574.2 nm when the concentration of the sucrose solution changed from 33% to 60%. The refractive indices of the sucrose solution with a concentration of 33%, 50%, and 60% were 1.39, 1.42, and 1.44, respectively. The refractive index of the sucrose solution was measured by using an Abbe refractometer (Rudolph Research Analytical, NJ, USA). The tuning rate of the polymer laser based on the SGA cavity near the refractive index value of 1.33 was 4.8 nm for per refractive index units (RIU). Figure 4b showed the tunability performance of the polymer laser based on the SAG cavity. When the ambient refractive index changed from 1.0 (air) to 1.33 (water), the emission wavelength shifted from 572.2 nm to 573.3 nm. The laser wavelength was tuned from 573.7 nm to 574.4 nm when the concentration of the sucrose solution changed from 33% to 60%. The tuning rate of the polymer laser based the SAG cavity near the refractive index value of 1.33 was approximately 3.3 nm/RIU. It can be seen that the polymer laser based on the SGA cavity is more sensitive than that based on the SAG cavity. Overall, the tuning range of the proposed cavity is small. To increase the tuning range, the cavity structures (period, film thickness, gain materials, and so on) should be optimized further.

**Figure 5:**
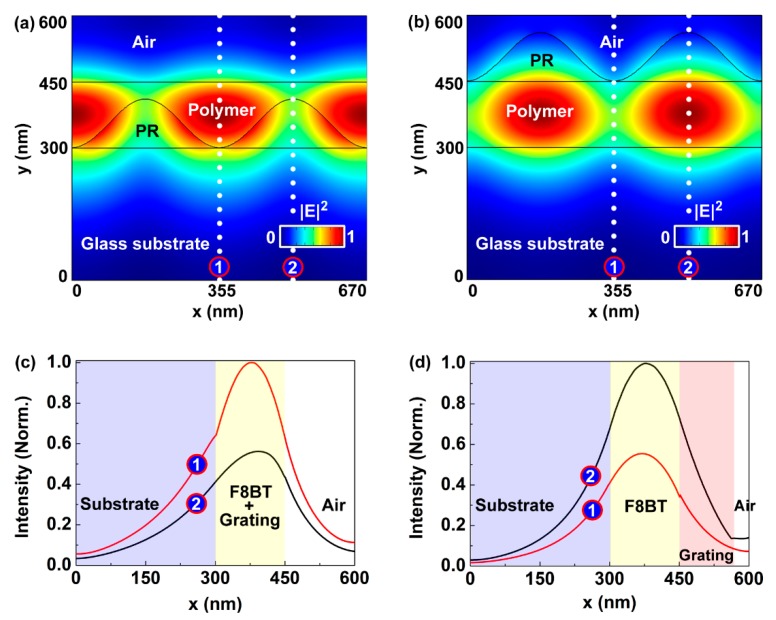
Electric field distribution of the 572 nm mode of (**a**) the SGA cavity and (**b**) the SAG cavity. Figures (**c**) and (**d**) are cross sections of the electrical field distribution of the mode extracted from (**a**) and (**b**) along the dotted white lines, respectively.

To study why the SGA cavity showed a relativity high tuning rate, the electric field distributions of the mode of the two kinds of cavities were simulated by using the COMSOL software, as shown in Figure 5. The refractive indices of the glass substrate, F8BT, and the photoresist around 572 nm were 1.51, 2.04 and 1.72, respectively, which were measured using an ellipsometer (ESNano, Ellitop Scientific, Bejing, China). The simulated parameters were the same as those shown in Figure 1. The model was built with a glass substrate (300 nm), a grating (110 nm), and a gain layer (150 nm), as shown in Figure 5a,b. It can be seen that the electrical field distribution of the 572 nm mode of the SGA cavity was much larger than that of the SAG cavity in the air region, which implied a large distribution of the laser mode in the liquid environment. For clarity, the cross sections of the electrical field distribution of the mode were demonstrated in Figure 5c,d, which were extracted from the electrical field distribution along the dotted white lines in Figure 5c,d, respectively. So, the mode volumes of the SGA cavity in the air region were 16% and 18% at position ① and ② in Figure 5a, respectively. In contrast, the mode volumes of the SAG cavity in the air region were 18% and 2% at positions ① and ②, respectively. It implied that the SGA cavity was sensitive to the ambient refractive index. So, the tunability of the SGA cavity was larger than that of the SAG cavity.

## 4. Conclusions

Two kinds of DFB cavities, the SGA and SAG structures, were employed to investigate the tuning properties of polymer lasers in the liquid environment. The emission wavelength of DFB polymer lasers can be tuned by changing the ambient refractive index of laser devices. The sucrose solutions with different concentrations were used to demonstrate the tunability of DFB polymer lasers. The DFB polymer lasers based on the SGA cavity showed a higher tunability than that based on the SAG cavity. This can be attributed to the fact that the field distribution of the laser mode of the SGA cavity was much larger than that of the SAG cavity in the liquid environment. These results may help to further explore light sources and sensors.
